# Efficacy of acupuncture based on acupoint combination theory for irritable bowel syndrome: a study protocol for a multicenter randomized controlled trial

**DOI:** 10.1186/s13063-021-05432-0

**Published:** 2021-10-19

**Authors:** Jing-wen Sun, Ming-liang Sun, Da Li, Jun Zhao, Su-hua Shi, Hui-xia Li, Hui-min Liu, Jun-xia Gao, Yu Hu, Hui Zheng, Xin Wang, Rong-dan Xue, Xue Feng, Shu-guang Yu, Zhi-gang Li

**Affiliations:** 1 School of Acupuncture-Moxibustion and Tuina, Beijing University of Chinese Medicine, Beijing, China; 2 Department of Endocrinology, Affiliated Hospital of Shandong University of Traditional Chinese Medicine, Jinan, China; 3 Department of Acupuncture and Moxibustion, Dongzhimen Hospital Affiliated with Beijing University of Chinese Medicine, Beijing, China; 4 Department of Rehabilitation, Third Affiliated Hospital of Beijing University of Chinese Medicine, Beijing, China; 5 Department of Gastroenterology, Third Affiliated Hospital of Beijing University of Chinese Medicine, Beijing, China; 6 Chengdu University of Traditional Chinese Medicine, Chengdu, Sichuan China; 7 Department of Acupuncture and Moxibustion, Beijing Hospital of Traditional Chinese Medicine, Capital Medical University, Beijing, China; 8 Department of Acupuncture and Moxibustion, Dongfang Hospital Affiliated with Beijing University of Chinese Medicine, Beijing, China; 9 Chinese Association of Chinese Medicine, Beijing, China

**Keywords:** Acupuncture, Irritable bowel syndrome, Acupoint combination, Randomized controlled trial, Protocol

## Abstract

**Background:**

Irritable bowel syndrome (IBS) is a chronic gastrointestinal disorder characterized by abdominal pain, diarrhea or constipation, and changes in defecation patterns. No organic disease is found to explain these symptoms by routine clinical examination. This study aims to investigate the efficacy and safety of acupuncture therapy for IBS patients compared with those of conventional treatments. We also aim to identify the optimal acupoint combination recommended for IBS and to clarify the clinical advantage of the “multiacupoint co-effect and synergistic effect.”

**Methods and analysis:**

A total of 204 eligible patients who meet the Rome IV criteria for IBS will be randomly stratified into acupuncture group A, acupuncture group B, or the control group in a 1:1:1 ratio with a central web-based randomization system. The prespecified acupoints used in the control group will include bilateral *Tianshu (ST25)*, *Shangjuxu (ST37)*, *Neiguan (PC6)*, and *Zusanli (ST36).* The prespecified acupoints used in experimental group A will include bilateral *Tianshu (ST25)*, *Shangjuxu (ST37)*, and *Neiguan (PC6)*. The prespecified acupoints used in experimental group B will include bilateral *Tianshu (ST25)*, *Shangjuxu (ST37)*, and *Zusanli (ST36)*. Each patient will receive 12 acupuncture treatments over 4 weeks and will be followed up for 4 weeks. The primary outcome is the IBS-Symptom Severity Scale (IBS-SSS) score. The secondary outcomes include the Bristol Stool Form Scale (BSFS), Work and Social Adjustment Score (WSAS), IBS-Quality of Life (IBS-QOL), Self-Rating Anxiety Scale (SAS), and Self-Rating Depression Scale (SDS) scores. Both the primary outcome and the secondary outcome measures will be collected at baseline, at 2 and 4 weeks during the intervention, and at 6 weeks and 8 weeks after the intervention.

**Ethics and dissemination:**

The entire project has been approved by the ethics committee of the Beijing University of Chinese Medicine (2020BZYLL0903).

**Discussion:**

This is a multicenter randomized controlled trial for IBS in China. The findings may shed light on the efficacy of acupuncture as an alternative to conventional IBS treatment. The results of the trial will be disseminated in peer-reviewed publications.

**Trial registration:**

Chinese Clinical Trials Register ChiCTR2000041215. First registered on 12 December 2020. http://www.chictr.org.cn/.

## Introduction

Irritable bowel syndrome (IBS) is a common functional gastrointestinal disorder characterized by abdominal pain associated with stool abnormalities and changes in stool consistency [1]. Epidemiological data show that the prevalence of IBS is approximately 11.2% worldwide [2], 5–6% in China [3], and approximately 10–20% in adults in Western countries [4], and the incidence is increasing yearly, which indicates that IBS is a major public health issue. The main symptoms of IBS are abdominal pain, abdominal distension, or abdominal discomfort, which are related to bowel movements or associated with changes in bowel habits such as frequency and/or fecal traits. Irritable bowel syndrome is a common medical condition that significantly alters the patient’s quality of life and presents a series of diagnostic and treatment challenges to the treating provider [5].

It is currently believed that genetic susceptibility, diet, abnormal intestinal motility, visceral hypersensitivity, intestinal inflammation and infection, intestinal flora disorders, neurological and neuroimmunological factors, mental and psychological factors, etc. are all related to the pathogenesis of IBS [6]. Despite many findings and advances in IBS research, the pathophysiology of IBS is still poorly understood, and there is no definitive biomarker, thereby hampering the development of effective therapies for IBS.

Recent studies have shown that there has been a rise in the use of nonpharmacological therapies, including acupuncture, for the treatment of IBS [7, 8]. As a traditional Chinese medicine treatment, acupuncture has been widely used to alleviate the resulting pain from IBS. In addition, acupuncture is widely accepted in clinical practice as an effective treatment option for gastrointestinal disorders. Acupuncture could be used as an adjunctive treatment in clinical settings to improve treatment efficacy. Relative to sham controls, acupuncture showed no superiority for treating IBS, while the advantage over Western medicine was significant [9]. Although there has been an increasing number of randomized controlled trials on traditional Chinese medicine treatment of IBS in recent years, and a few of them have observed adverse reactions, a meta-analysis showed that there is considerable heterogeneity among the studies, and no significant difference conclusions can be obtained [10]. Therefore, future high-quality and large-sample-size studies with adequate quantity-effect designs need to be conducted. As a result, our team has designed a multicenter, large-sample, double-blind randomized controlled trial to explore a relatively suitable acupoint combination and to provide certain clinical evidence for the optimization of acupuncture treatment of IBS.

## Methods/design

**Fig. 1:**
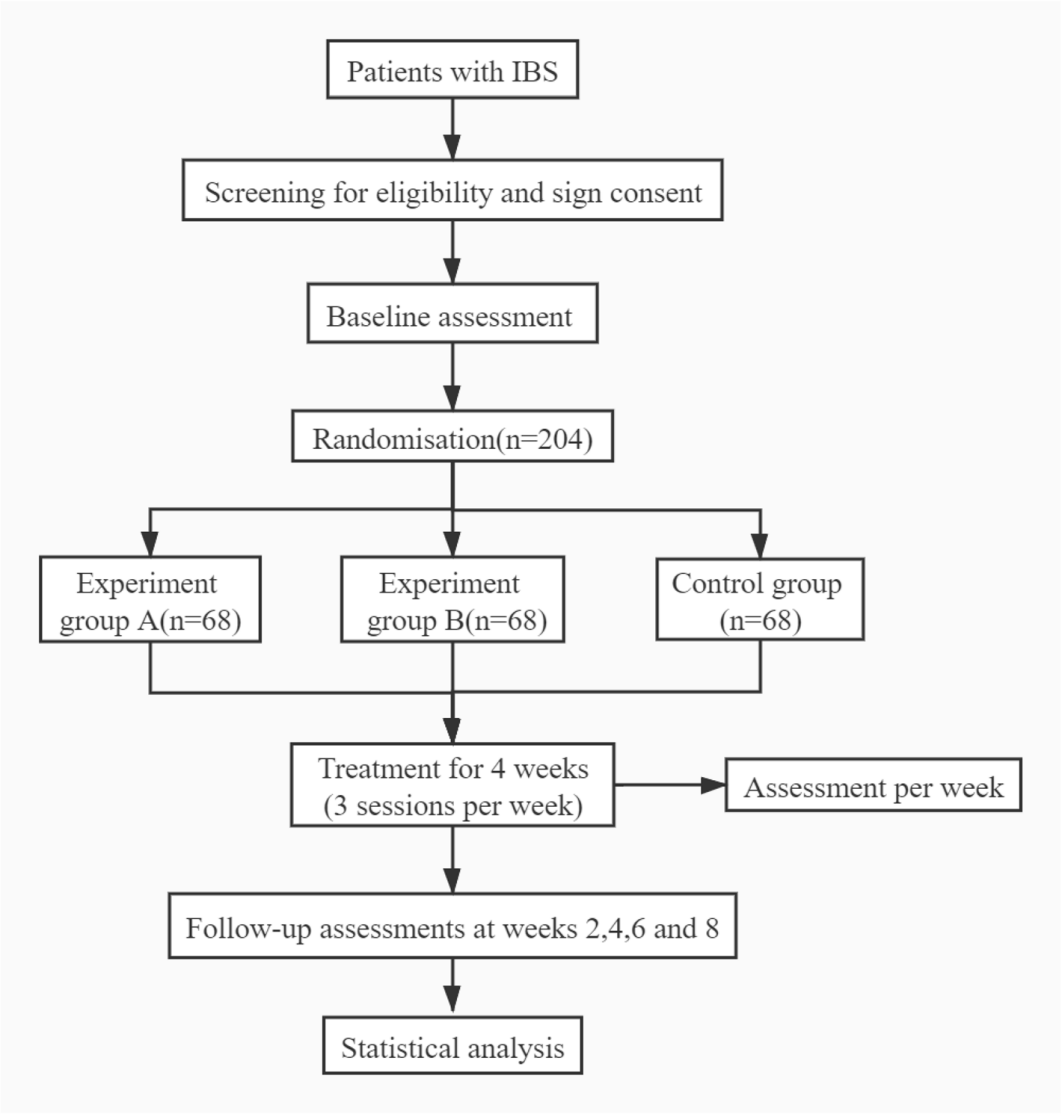
Flow diagram

The protocol for this trial is reported based on the Standard Protocol Items: Recommendations for Interventional Trials (SPIRIT) 2013 Checklist [11]: defining standard protocol items for clinical trials. This multicenter randomized controlled trial will be conducted at 6 hospitals in China: Dongzhimen Hospital Affiliated with Beijing University of Chinese Medicine; Dongfang Hospital Affiliated with Beijing University of Chinese Medicine; Third Hospital Affiliated with Beijing University of Chinese Medicine; Beijing Hospital of Traditional Chinese Medicine Affiliated with Capital Medical University; Clinical Medicine College/Teaching Hospital Affiliated with Chengdu University of Traditional Chinese Medicine; and Third Hospital/Acupuncture and Tuina School Affiliated with the Chengdu University of Chinese Medicine. The study was approved by ethics committees and registered at www.chictr.org.cn on 14 December 2020 (Registration number: ChiCTR2000041215). The eligible patients will be randomly divided into three groups, and participants will receive a 1-week wash-out, 12 sessions over 4 weeks of treatment, and 4 weeks of posttreatment follow-up. Outcomes will be assessed at baseline, during the treatment, and at the end of the follow-up. Figure 1 illustrates the flow diagram of the trial.

### Patient recruitment

Patients meeting the Rome IV IBS criteria will be primarily recruited from the outpatient centers of the 6 participating hospitals and their surrounding communities. Other methods of recruitment include advertisements on hospital social media (WeChat subscription, Tik Tok, etc.), posters, flyers about this trial, and outpatient clinics. Clinicians will primarily select patients with suspected IBS for further detailed assessment. One clinical research assistant will identify eligible participants for this trial according to the following rigorous inclusion and exclusion criteria. All patients will voluntarily agree to participate and sign an informed consent document before randomization.

The Rome IV criteria include the following [12]: (1) Diagnostic time frame: ① ≥ 3 months of persistent symptoms with symptom onset at least 6 months before diagnosis and ② symptoms ≥ 1 day per week; (2) Symptom description: abdominal pain; (3) Symptoms recurrently associated with 2 or more of the following criteria: ① symptoms are related to defecation, ② symptoms are associated with a change in stool form, and ③ symptoms are associated with a change in the frequency of defecation.

The following inclusion criteria will be used: (1) age 18–70 years, (2) fulfillment of the Rome IV criteria for IBS, (3) symptoms present for ≥ 6 months, (4) baseline IBS-Symptom Severity Scale (IBS-SSS) score of 75 points or greater, (5) no abnormal occult blood in the past month, (6) no acupuncture treatment for 3 months preceding the trial, and (7) agreement to participate and sign the informed consent document.

The following exclusion criteria will be used: (1) patients with past colonoscopy, barium meal fluoroscopy, abdominal ultrasound, and other examinations that found organic intestinal diseases (including but not limited to patients with ulcerative colitis and colorectal cancer); (2) patients with one or more of the following warning symptoms: blood in the stool, fecal occult blood test positive, anemia, abdominal mass, ascites, fever, and weight loss; (3) patients with serious diseases of the cardiovascular system, endocrine system, or rheumatic immune system that affect the judgment of the condition; (4) patients who are unconscious, unable to express subjective symptoms of discomfort or have a clear diagnosis of a severe mental disorder; (5) patients who are pregnant, lactating, and have a postpartum period of fewer than 12 months; and (6) patients who have received acupuncture for IBS in the past three months. The physical examination will be conducted at the time of recruitment to exclude participants with serious diseases who are not suitable for trial participation.

### Randomization and blinding

A blocked randomization sequence for multiple centers will be generated by an independent professional statistician (Linkermed Tech Co., Ltd.) using Statistical Analysis System software. All eligible participants will be randomly stratified into acupuncture group A, acupuncture group B, or the control group in a 1:1:1 ratio with a central web-based randomization system. Outcome evaluators and statisticians will not participate in the treatment but will conduct outcome evaluation and statistical analysis independently. Both patients and assistants will be blind to group assignments. However, due to the nature of acupuncture, the acupuncturist who provides the intervention will not be blinded. To minimize the known bias sources, the location of the acupuncture points and manipulation of needles will be similar in acupuncture group A, acupuncture group B, and the control group, thereby optimizing the blinding of subjects.

### Interventions

To ensure the safety and proper operation of acupuncture, all acupuncturists will participate in the standard operation process (SOP) of skin disinfection, acupoint location, fundamental puncture, and needle manipulations before the trials.

**Fig. 2:**
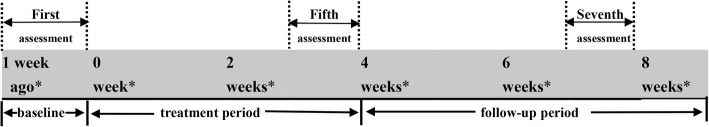
Stages of research

**Fig. 3:**
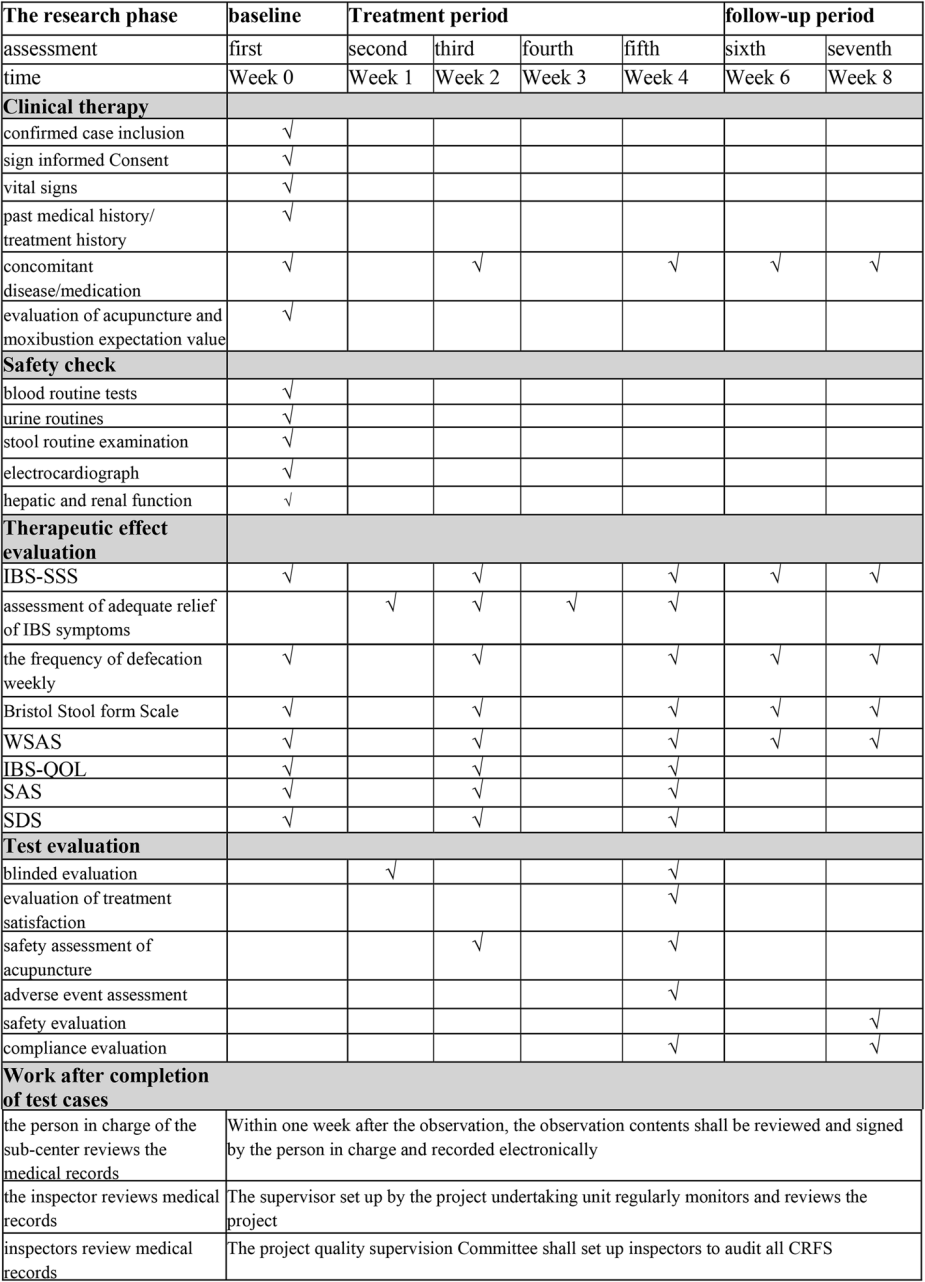
Schedule of enrollment, intervention, and assessments of this study protocol

Treatments will be performed by licensed acupuncturists who have at least 3 years of experience in acupuncture. Acupuncture will be discontinued if the patients suffer from any adverse events (AEs) after acupuncture. The stages of research are shown in Fig. 2. The schedule of enrollment, interventions, assessments, and participant visits is shown in Fig. 3.

### Treatment as usual (TAU)

TAU is defined as the continuation of current therapeutic agents prescribed by a general practitioner or gastroenterologist. All patients will continue treatment as usual during the study period. Any changes in personal medication will be recorded on a diary card.

### The experimental group and the control group

**Fig. 4:**
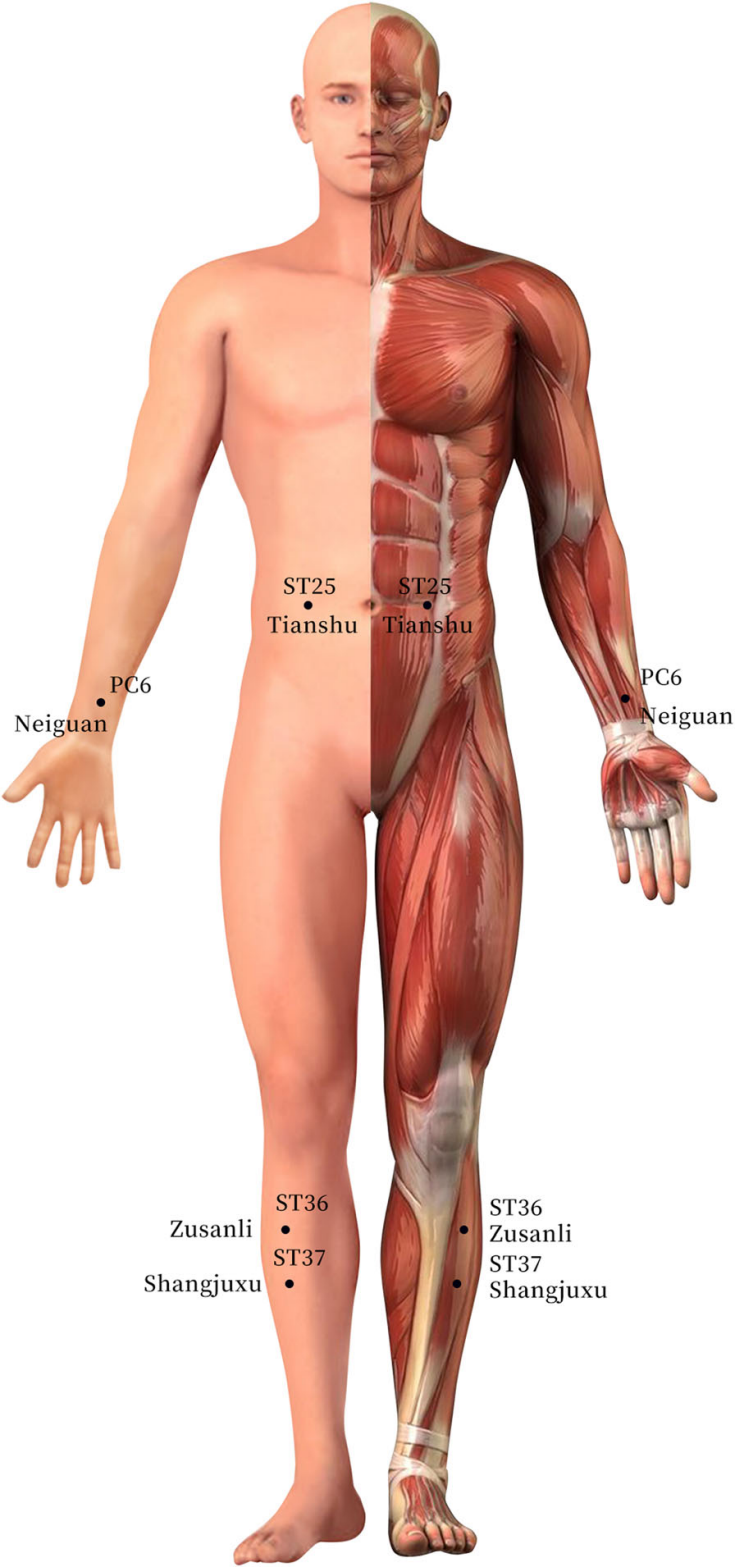
Locations of acupoints

Patients allocated to either the experimental group or the control group will receive acupuncture treatment (with disposable and sterile acupuncture needles; length: 25–40 mm, diameter: 0.30 mm; Huatuo, Suzhou, China) three times a week for 4 weeks for a total of 12 treatment sessions. Based on traditional Chinese medicine theory and our clinical experience, the prespecified acupoints used in the control group will include bilateral *Tianshu (ST25)*, *Shangjuxu (ST37)*, *Neiguan (PC6)*, and *Zusanli (ST36).* The prespecified acupoints used in experimental group A will include bilateral *Tianshu (ST25)*, *Shangjuxu (ST37)*, and *Neiguan (PC6).* The prespecified acupoints used in experimental group B will include bilateral *Tianshu (ST25)*, *Shangjuxu (ST37)*, and *Zusanli (ST36)* (see Tables 1, 2, and 3 and Fig. 4).

**Table 1:** Locations of acupoints in the control group

Acupoints	Locations
Tianshu (ST25)	On the same level of the umbilicus, and 2 cun* lateral to the anterior midline
Shangjuxu (ST37)	6 cun directly below Dubi (ST35), and one finger-breadth lateral to the anterior border of the tibia
Neiguan (PC6)	On the line joining Daling (PC7) and Quze (PC3), between the tendons of the palmaris longus and flexor carpi radials, 2 cun above the transverse crease of the wrist
Zusanli (ST36)	3 cun directly below Dubi (ST35), and one finger-breadth lateral to the anterior border of the tibia

*1 cun (≈20 mm) is defined as the width of the interphalangeal joint of the patient’s thumb

**Table 2:** Locations of acupoints in experiment group A

Acupoints	Locations
Tianshu (ST25)	On the same level of the umbilicus, and 2 cun* lateral to the anterior midline
Shangjuxu (ST37)	6 cun directly below Dubi (ST35), and one finger-breadth lateral to the anterior border of the tibia
Neiguan (PC6)	On the line joining Daling (PC7) and Quze (PC3), between the tendons of the palmaris longus and flexor carpi radials, 2 cun above the transverse crease of the wrist

*1 cun (≈20 mm) is defined as the width of the interphalangeal joint of the patient’s thumb

**Table 3:** Locations of acupoints in experiment group B

Acupoints	Locations
Tianshu (ST25)	On the same level of the umbilicus, and 2 cun* lateral to the anterior midline
Shangjuxu (ST37)	6 cun directly below Dubi (ST35), and one finger-breadth lateral to the anterior border of the tibia
Zusanli (ST36)	3 cun directly below Dubi (ST35), and one finger-breadth lateral to the anterior border of the tibia

*1 cun (≈20 mm) is defined as the width of the interphalangeal joint of the patient’s thumb

The exact location and depth of needling for each point will be determined based on the 2006 People’s Republic of China National Standard: The Name and Location of Acupoints (GB/T 12346-2006) [13]. After insertion, all points will be manually stimulated by twirling and lifting the needle every 10 min to elicit “deqi”, which is characterized by distention, soreness, numbness, etc. In addition, the needles will be retained for 30 min.

## Outcomes

### Primary outcome

#### IBS-Symptom Severity Scale (IBS-SSS) score

Several questionnaires exist to assist in the diagnosis and assessment of the severity of the disease. One of these is the Irritable Bowel Syndrome-Severity Scoring Scale (IBS-SSS). The English version of the IBS-SSS was validated in 1997 [14]. The questionnaire is scored from 0 to 500 and is composed of five 100-point scales that measure the severity of pain (IBS-SSS1), the frequency of abdominal pain (IBS-SSS2), the severity of abdominal distention (IBS-SSS3), satisfaction with bowel habits (IBS-SSS4), and disruption in quality of life (IBS-SSS5). The equation for the calculation of abdominal pain day points is as follows: abdominal pain day points = (days of abdominal pain in 7 days/7) × 100. The IBS-SSS total score = the number of days of abdominal pain + the score of abdominal pain + the score of abdominal distension + the score of defecation satisfaction + the score of impact on life, where the possible score on each item is 100 points, with a total score possible of 500 points, and higher scores indicate more severe illness. The scores are classified into normal (< 75), mild IBS (75–175), moderate IBS (175–300), and severe IBS (> 300), and these scores will be used as the primary outcome in this trial. IBS-SSS score will be assessed at baseline and at 2, 4, 6, and 8 weeks.

### Secondary outcomes

#### Bristol Stool Form Scale

The Bristol Stool Form Scale (BSFS) is a 7-point scale used extensively in clinical practice and research for stool form measurement [15]. In this trial, participants will be asked to record the type and score of defecations in a week. The scores obtained from the Bristol scale will be assessed at baseline and weeks 2, 4, 6, and 8. Stool traits will be classified using a score of 1 point for type 1, 2 points for type 2, and so on; for participants with multiple bowel movements in a day, the highest score will be recorded.

#### Work and Social Adjustment Scale (WSAS)

The Work and Social Adjustment Scale (WSAS) was designed to measure patients' perceived functional impairment associated with a health problem [16]. The Work and Society Adjustment Scale measures the impact of IBS on a patient’s family, work and management abilities; participation in social and private leisure activities; and maintenance of interpersonal relationships. The WSAS is composed of five items, and each item is scored from 0 to 8 points. Zero points means no impact, and 8 points means severe impact. Finally, the total score of the scale is calculated. The WSAS will be assessed at baseline and weeks 2, 4, 6, and 8.

#### IBS-Quality of Life (IBS-QOL)

The IBS-QOL items demonstrate very good construct validity and the ability to detect changes due to treatment effects [17]. The questionnaire consists of 34 items. Each item includes a 5-point response scale. Patients will complete the IBS-QOL questionnaires at baseline, 2 weeks, and 4 weeks after treatment. The total scores on each item will be calculated and analyzed, with higher scores suggesting better QOL.

#### Self-Rating Anxiety Scale (SAS) and Self-Rating Depression Scale (SDS)

The Self-Rating Anxiety Scale (SAS) is a norm-referenced scale that is widely used in screening for anxiety disorders [18]. The Self-Rating Depression Scale (SDS) is an established norm-referenced screening measure used to identify the presence of depressive disorders in adults [19]. IBS-M is more likely to be associated with a higher level of depression and anxiety, and the prevalence of depression and anxiety in IBS-C is highest. Psychological screening and appropriate psychotherapy are needed for patients with IBS-C, IBS-D, and IBS-M [20]. The SAS and SDS each consist of 20 items, and each item has four options corresponding to 1–4 points. Finally, the total score of the scale is calculated. An SDS total score of 53–62 indicates mild depression, 63–72 indicates moderate depression, and greater than 72 indicates severe depression. An SAS total score of 50–60 indicates mild anxiety, 61–70 indicates moderate anxiety, and greater than 70 indicates severe anxiety. Therefore, anxiety and depression symptoms will be assessed using the SAS and SDS at baseline, week 2, and week 4. Higher scores indicate higher levels of anxiety and depression.

### Blinding assessment

To test the patient-blinding effects, all patients will be asked to guess which group they were assigned to at the end of the first treatment session and the twelfth treatment session.

### Safety evaluation

The research assistant will be required to document all the AEs on the Case Report Form (CRF), including information such as the time of occurrence, severity, duration, measurement, management, and outcome. During the trial, adverse events will be monitored during the 4 weeks of treatment and the next 4 weeks of follow-up. The common side effects of acupuncture include subcutaneous hematoma, local bleeding, skin bruising, pruritus or persistent pain at the acupuncture site, muscle spasm, dizziness, and so on. When these conditions occur, they should be recorded in detail, including the time of occurrence, symptoms, signs, severity, duration, laboratory test indicators, treatment methods and results, elapsed time, and follow-up time for adverse reactions. Patients should be properly managed until they are completely normal. In the event of a serious adverse event (SAE), the necessary action will be taken immediately for the subject's safety. The clinician will report to the Beijing University of Traditional Chinese Medicine Key Research and Development Project 4 Management Office within 24 h when the subject discovers an adverse reaction. The data and safety supervision committee will decide whether to terminate the test. The clinician executes the orders of the data and safety supervision committee. The Department of Acupuncture and Moxibustion of the six abovementioned hospitals will be responsible for the treatment of all AEs.

### Sample size and statistical analysis

Based on previous clinical experience, we anticipate an improvement in the IBS-SSS score of 30 points in experiment group A and experiment group B and 35 points in the control group. The standard deviation is set at 10, and the ratio among experiment group A, experiment group B, and control group C is 1:1:1. The sample size was calculated with a 15% type II error rate (80% power) and 5% type I error. A superiority trial will be adopted to minimize differences among the three groups, and a score of 10 points is expected. In total, a sample size of 177 (59 patients in each group) was estimated. Allowing for 10% loss to follow-up, the sample size will be expanded to 68 patients in each group.

All analyses will be performed using the intent-to-treat (ITT) and the per-protocol (PP) populations. ITT analysis requires all participants to receive a baseline assessment of the primary outcome and at least 1 acupuncture session. Missing values will be imputed through the multiple imputation method. The PP population is usually defined as patients who complete at least 80% of the treatment protocol without major protocol violations. The primary outcome will be assessed using analysis of covariance (ANCOVA) and adjusted for baseline total IBS-SSS score. For other secondary outcomes, continuous variables will be analyzed using Student’s *t* test or Wilcoxon rank-sum test, and data will be represented as the mean ± SD or median. Categorical variables will be calculated using Fisher’s exact test or Wilcoxon rank-sum test, and data will be represented by the frequency (percentage). In terms of efficacy factors, other covariates, such as age, sex, disease duration, and conventional pharmacological agent classification, will be considered for further analyses. The differences in blinding and adverse events between the groups will be compared using the chi-square test. In addition, we decided to carry out subgroup analyses based on IBS subtypes, avoiding a heterogeneous patient population which may affect the efficacy results.

Statistical analysis of all data in this study will be performed by a specialized third-party statistical evaluation. SAS, version 8.2 statistical software (SAS Institute Inc.) will be used for data analysis. A two-sided *P* < 0.05 will be considered statistically significant.

### Data management and quality control

To ensure the objectivity of the data, the data of individual participants will not be disclosed externally. All researchers will receive training on data management. Data input and management will be carried out by the data management staff of the Chinese Academy of Chinese Medical Sciences using the database software clinical trial data management system. The electronic case report form (e CRF) will be designed to collect all clinical observations of each participant before recruitment. The research assistant will be responsible for verifying the accuracy and completeness of the data to prevent any errors or omissions and for ensuring that items do not need to be changed or clarified. After the research is completed, the data administrator will implement a data lock. All paper documents and electronic data will be kept for 5 years after publication and will be destroyed afterward.

To ensure the quality of the trial, a prespecified SOP, including information on the recruitment and screening of participants, randomization, laboratory detection results, intervention, details on completing the CRF, the assessment of outcomes, data management, and AEs of acupuncture, will be unified and trained through a multicenter internal study for the consistency of results. In addition, we will set up a data and security monitoring board, an independent advisory group, to review and explain the data generated by the research, and we will hold meetings every three months to report on the progress of the research. Each amendment of the protocol conforms to the GCP principles and maintains the ethical standards of the RCT.

To ensure the reliability of the test results, the quality of the research, and the protection of the rights and interests of trial subjects, a data and safety supervision committee was established to systematically supervise every step of the experiment. The main investigator, investigator, and all clinical trial supervisors must carry out this operation procedure. The person in charge of each district and the director of each hospital shall be responsible for supervision and inspection, and the main investigator shall be responsible for random inspection of the implementation.

### Patients and public involvement

Patients in this trial will not be involved in the design or conduct of the study or the outcome assessments. However, we are planning to disseminate our research to the participants and the public, for example, by publicizing our research on hospital social media (WeChat subscription, Tik Tok app) and presenting our findings at various academic lectures. We will report all adverse events in the trial publication.

### Ethics and dissemination

This study complies with the principles of the Declaration of Helsinki and relevant ethical guidelines. The entire project has been approved by the ethics committee of Beijing University of Chinese Medicine (ID:2020BZYLL0903). Written informed consent will be obtained from patients before enrollment in the study. The outcomes of the trial will be disseminated through peer-reviewed publications.

## Discussion

Irritable bowel syndrome (IBS) is a common nonorganic gastrointestinal disorder. However, the etiology of the disease is unknown, and there is no effective treatment. IBS is a large economic burden to both the patients themselves and society, and IBS can adversely affect quality of life, even though IBS does not increase mortality.

As a traditional Chinese medicine external treatment method, acupuncture is characterized by its simple operation, significant treatment effects, and few side effects. Acupuncture has been widely used in treating diarrhea-predominant irritable bowel syndrome (IBS-D) [21]. Compared with routine pharmacotherapies and placebo, acupuncture and cognitive-behavioral therapy (CBT) had better efficacy in relieving IBS symptoms. Based on the SUCRA values, acupuncture ranked first in improving overall clinical efficacy and avoiding adverse effects [22]. Acupuncture produced a more significant effect than loperamide in weekly defecation. Compared with dicetel, acupuncture showed more effectiveness in terms of IBS-Symptom Severity Scale scores. In addition, compared to dicetel and trimebutine maleate, acupuncture also showed more effectiveness in terms of total efficiency. Acupuncture treatment has been shown to improve the clinical effectiveness of IBS-D or FD, with great safety, but the conclusions of this study need to be further verified with a higher quality of evidence [23]. Acupuncture and electroacupuncture (low/very low-level evidence) may benefit constipation. The next 20 years should be exciting in the field as higher-level studies are performed [24].

Acupoint combination, which is based on traditional Chinese medicine theories, is now widely used in clinical treatment. From the perspective of traditional Chinese medicine, the combination of acupoints based on syndrome differentiation can integrate the specific effects of different acupoints to produce synergistic effects for better clinical efficacy. Hence, acupoint combination is considered to be an important part of acupuncture prescription and the basis of acupuncture manipulation.

The use of acupoints in combination rather than a single acupoint is often the preferred treatment. Acupoint combination therapy, based on clinical experience, has a better curative effect and wider therapeutic scope. As the classic acupoint combination, the combination of the He and Mu acupoints has been proven effective and has a synergistic effect on functional dyspepsia [25]. Different acupoint combinations have specific synergistic effects that can be applied to relevant diseases. Therefore, the use of the most appropriate acupoint combination plays a critical role in the therapeutic effects of acupuncture. However, the efficacy of the acupoint combination should be investigated further in clinical trials due to a lack of a clear scientific explanation. As a consequence, we believe that a multicenter randomized controlled clinical trial that describes the different acupoint combinations of treating IBS is needed to identify the optimal clinical acupoint combination [26].

Based on previous research and our previous review, the basic acupoints that play an important role in relieving diarrhea symptoms are *Tianshu (ST25)*, *Zusanli (ST36)*, and *Shangjuxu (ST37)* [27]. Acupuncture highlights the specific functions of acupoints in the treatment of psychosomatic diseases. Clinical evidence shows that *Neiguan (PC6)* can improve anxiety symptoms very well [28]. Therefore, the selected acupoints of *Tianshu (ST25)*, *Zusanli (ST36)*, *Shangjuxu (ST37)*, and *Neiguan (PC6)* in this experiment not only accord with the principle of “treatment based on syndrome differentiation” in traditional Chinese medicine but also accord with the clinical practice guidelines in China. Therefore, there is an urgent need for a large-sample, multicenter randomized controlled trial to evaluate the efficacy of acupoint combinations in improving IBS symptoms within an evidence-based medicine framework.

This study aims to evaluate the efficacy and safety of acupuncture treatment for IBS by comparing different groups of acupoints. Based on conventional treatment, with *Tianshu (ST25)* and *Shangjuxu (ST37)* as the basic prescriptions, comparing *Tianshu (ST25)* and *Shangjuxu (ST37)* with *Neiguan (PC6)* or *Tianshu (ST25)* and *Shangjuxu (ST37)* with *Zusanli (ST36)* for the treatment of IBS clinical effect value, we want to clarify the clinical advantage of a “multiacupoint co-effect and synergistic effect.” The findings of the study will provide the optimal acupoint combination and high-quality clinical evidence for the compatibility of different acupoints in the treatment of IBS, facilitating clinical practice and further scientific studies.

Nevertheless, there are still limitations in this study that are worth noting. First, the primary outcome measure is a subjective rating scale score and therefore does not provide objective evidence to directly support the effectiveness of a particular treatment. Second, the drop-out rate is likely to be higher at the end of the 6-week treatment followed by a 3-month follow-up. To reduce the likelihood of dropout, we have developed guidelines and training for researchers and will establish good communication and relationships with participants.

In conclusion, we hope that the results of this study not only provide more reliable clinical evidence for the efficacy and safety of acupuncture in the treatment of irritable bowel syndrome but also demonstrate the value of clinical acupoint combinations in the treatment of irritable bowel syndrome.

## Data Availability

After the study is completed, all relevant data will be shared by the corresponding author upon reasonable request. This is an open-access article distributed by the Creative Commons Attribution Non-Commercial (CC BY- NC 4.0) license, which permits others to distribute, remix, adapt, build upon this work noncommercially, and license their derivative works on different terms, provided the original work is properly cited, appropriate credit is given, any changes made indicated, and the use is noncommercial. See http://creativecommons.org/licenses/by nc/4.0/.

## Abbreviations

IBS: Irritable bowel syndrome
IBS-SSS: IBS-Symptom Severity Scale
BSFS: Bristol Stool Form Scale
WSAS: Work and Social Adjustment Scale
IBS-QOL: IBS-Quality of Life
SAS: Self-Rating Anxiety Scale
SDS: Self-Rating Depression Scale
AE: Adverse event
SOP: Standard operation process
TAU: Treatment as usual
CRF: Case Report Form
SAE: Serious adverse event
IBS-D: Diarrhea-predominant irritable bowel syndrome
CBT: Cognitive-behavioral therapy
FD: Functional disease
